# A novel DNA/histone H4 peptide complex detects autoantibodies in systemic lupus erythematosus sera

**DOI:** 10.1186/s13075-016-1117-8

**Published:** 2016-10-04

**Authors:** Filomena Panza, Maria Claudia Alcaro, Fiorella Petrelli, Francesca Angelotti, Federico Pratesi, Paolo Rovero, Paola Migliorini

**Affiliations:** 1 Department of Clinical and Experimental Medicine, University of Pisa, Via Roma 67, 56126 Pisa, Italy; 2 Toscana Biomarkers Srl, Siena, Italy; 3 Laboratory of Peptide and Protein Chemistry and Biology, Department of Neurosciences, Psychology, Drug Research and Child Health, Section of Pharmaceutical Sciences and Nutraceutics, University of Florence, Via Ugo Schiff 6, 50019 Sesto Fiorentino, Italy; 4 Present address: DIESSE Diagnostica Senese SPA, via Fiorentina 1, 53100 Siena, Italy

**Keywords:** Anti-DNA antibodies, Systemic lupus erythematosus, Synthetic peptides, Autoantibodies, Autoimmune diseases

## Abstract

**Background:**

The detection of anti-dsDNA antibodies is critical for the diagnosis and follow-up of systemic lupus erythematosus (SLE) patients. The presently available assays are characterized by a non-optimal specificity (solid phase assays) or sensitivity (*Crithidia Luciliae* immunofluorescence test (CLIFT)). To overcome the limits of CLIFT and solid phase chromatin assays, we explored the diagnostic potential of an assay based on plasmid DNA containing a highly bent fragment of 211 bp from *Crithidia Luciliae* minicircles, complexed with histone peptides.

**Methods:**

Electrically neutral complexes of PK201/CAT plasmid (PK) DNA and histone 4 (H4) peptides were evaluated by electromobility shift assay. Complexes of H4 peptides and PK were absorbed to the solid phase to detect specific immunoglobulin G (IgG) in sera. Sera from 109 SLE patients, 100 normal healthy subjects, and 169 disease controls were tested.

**Results:**

H4(14-34) containing the consensus sequence for DNA binding interacts with PK, retarding its migration. H4(14-34)/PK complexes were used to test sera by ELISA. Anti-H4-PK antibodies were detected in 56 % of SLE sera (more frequently in patients with skin or joint involvement) versus 5.9 % in disease controls; inhibition assays show that sera react with epitopes present on DNA or on the complex, not on the peptide. Antibody titer is correlated with European Consensus Lupus Activity Measurement (ECLAM) score and anti-complement component 1q (C1q) antibodies, negatively with C3 levels. Anti-H4-PK antibodies compared with CLIFT and solid phase dsDNA assays display moderate concordance.

**Conclusions:**

The H4/PK assay is a simple and reliable test which is useful for the differential diagnosis and evaluation of disease activity in SLE patients.

**Electronic supplementary material:**

The online version of this article (doi:10.1186/s13075-016-1117-8) contains supplementary material, which is available to authorized users.

## Background

Anti-dsDNA antibodies react with linear and conformational determinants exposed on the double helix of DNA and cross-react with phospholipids, carbohydrates, and proteins [1]. These autoantibodies are exclusively detected in sera from patients affected by systemic lupus erythematosus (SLE) and in animal models of the disease [2–4]. Thus, the presence in sera of anti-dsDNA antibodies is one of the serological criteria for the diagnosis of SLE [5, 6]. It is also well known that the titer of anti-dsDNA antibodies fluctuates with disease activity and is related to disease flares, especially at the renal level [7, 8]. Because of this diagnostic and prognostic relevance, simple and reliable assays for their detection are needed [9]. A currently used assay for anti-dsDNA antibodies is the immunofluorescence test that detects immunoglobulin G (IgG) antibodies binding the circular DNA in kinetoplasts of *Crithidia Fasciculata var. Luciliae* (CLIF test) [10]. The kinetoplast DNA has one of the highest degrees of stable curvature, resembling nucleosomal DNA, and it has been proposed that antibodies detected by CLIF are probably reactive with nucleosomes in vivo [11, 12].

It is well known that the CLIF test (CLIFT) is highly specific for the diagnosis of SLE but poorly sensitive; positivity in the assay is fairly predictive of active disease, especially at the renal and hematological level [13, 14]. Another criticism of the CLIFT is inherent to the performance of immunofluorescent assays, which require trained personnel and give semi-quantitative results. Because of these limits, a number of solid phase assays for the detection of anti-dsDNA antibodies have been proposed and commercialized. These assays differ widely for a number of parameters, including the source of DNA (genomic or plasmidic), the technique to absorb DNA to the solid phase, the type of solid phase, and the detection system. In parallel with this heterogeneity, the performance of ELISA is variable; using normal blood donors as controls and setting specificity at 95 %, the sensitivity can vary between 60 and 80 %. More differences are detected when sera from patients affected by other autoimmune disorders are evaluated. In this setting, the ability of ELISA to discriminate SLE from other disorders can be poor [13, 14].

Similar observations are applicable to anti-nucleosome antibodies, a family of anti-chromatin antibodies, measured by solid phase assays using intact or H1-stripped nucleosomes that detect antibodies reactive with DNA, histones, or determinants formed by the association of DNA with histones [15, 16]. Anti-nucleosome antibodies display a sensitivity and specificity similar to solid phase assays for anti-dsDNA antibodies, and similar correlations with disease activity and organ involvement in SLE. However, anti-nucleosome antibodies are detected also in patients with other connective tissue disorders, and namely in systemic sclerosis, mixed connective tissue disorder, and primary anti-phospholipid syndrome [17]. Thus, they represent a valuable tool for the analysis of SLE patients, but are not optimal in the differential diagnosis of SLE versus other systemic autoimmune disorders.

To overcome the limits of CLIFT and solid phase chromatin assays, we explored the diagnostic potential of an assay based on plasmid DNA containing a highly bent fragment of 211 bp from *Crithidia Luciliae* minicircles [18], complexed with histone peptides. As the interaction of histone 4 (H4) with DNA has been finely mapped [19, 20], H4 peptides containing the consensus sequence for DNA binding were selected and synthesized. A specific and sensitive assay was obtained that detects antibodies exclusively in SLE sera and gives complementary results when compared with CLIFT and ELISA.

## Methods

### Patients

A cohort of 109 SLE patients (99 female and 10 males, aged 15–71 years, mean age 34 years) attending the Clinical Immunology and Rheumatology Units of the University of Pisa were included in this study. Two samples were obtained from 16 patients (more than 1 month apart) and, on the whole, 125 sera were analyzed.

A full clinical and serological evaluation was performed that included measurement of complement levels, anti-dsDNA, and anti-complement component 1q (C1q) antibodies. Anti-dsDNA antibodies were detected by a commercial ELISA (Aeskulisa, Aesku Diagnostics, Wendelsheim, Germany), according to the manufacturer’s instructions, and by CLIFT. Anti-C1q antibodies were detected by ELISA as previously described [21]. On the basis of clinical and serological findings, a disease activity score (European Consensus Lupus Activity Measurement; ECLAM) [22] was calculated; an ECLAM score >2 was considered to be indicative of active disease.

One hundred and sixty-nine patients (151 females, 18 males) affected by other systemic autoimmune disorders were also enrolled (40 rheumatoid arthritis (RA), 30 idiopathic inflammatory myopathies (IIM) (9 polymyositis (PM) and 6 dermatomyositis (DM)), 29 undifferentiated connective tissue disease (UCTD), 27 Sjogren syndrome (SjS), 29 systemic sclerosis (SSc), 5 anti-phospholipid syndrome (APS), 4 psoriasic arthritis (PsA), 3 polymyalgia rheumatica (PMR), and 2 systemic vasculitis). The diagnosis of SSc was based on the ACR criteria [23]; PsA was diagnosed according to the criteria of Vasey and Espinoza [24]; SjS was diagnosed according to the criteria of the American European Consensus Group [25]; UCTD patients were classified as stable UCTD, with a disease duration longer than 5 years [26]; RA was diagnosed according to ACR/EULAR criteria 2010 [27]; and PM/DM were diagnosed according to Bohan and Peter [28].

One hundred normal healthy subjects (NHS) served as controls. Informed consent was obtained from all the subjects, and the study was approved by the local Ethical Committee (protocol 3661/2012).

### Peptide synthesis

Peptides were obtained by solid phase synthesis and purified to homogeneity by semi-preparative HPLC, according to previously reported methods optimized in our laboratory [29]. Their sequences and some relevant chemical properties are shown in Table 1.

**Table 1:** Sequences, isoelectric points (pI), and DNA consensus sequence (written in bold) of the histone peptides used in the study

Name	Sequence	pI	DNA consensus sequence
H2B(107-125)	AKHAVSEGTKAVTKYTSSK	9.83	n.a.
H4(56-71)	GVLKVFLENVIRDAVT	6.07	0
H4(76-91)-Gly	AKRKTVTAMDVVYALKG	10.00	0
H4(7-22)	GKGLGKGGA**KRHRK**VL	12.03	KRHRK
H4(31-50)	KPAIRRLARRGGVKRISGLI	12.6	0
H4(14-34)	GA**KRHRK**VLRDNIQGITKPAI	11.73	KRHRK
H4(21-37)	VLRDNIQGITKPAIRRL	11.71	0
MAP-Cys-[H4(14-34)]4	H4-(GA**KRHRK**VLRDNIQGITKPAI)4-Lys2-Lys-bAla-Cys-OH	12.16	KRHRK
H4(14-22)	GA**KRHRK**VL	12.02	KRHRK
Scramble H4(14-34)	KIVPKTLHRGDNRAKQGIRAI	11.73	0
FcεRIα(46-65)	SEETNSSLNIVNAKFEDSGE	3.91	Not present

*H4* histone 4

### Cloning of pDNA in competent *Escherichia coli* cells and pDNA extraction

The high-copy PK201/CAT plasmid, a kind gift of E. Di Mauro (University of Rome, “La Sapienza”), is 3228 base pairs long and contains the *Stu*I–*Acc*I 211-bp bent segment from the Trypanosomatidae protozoan *C. fasciculata* cloned in the *Bam*HI site of the vector pSP65 [18].

*E. coli* competent cells (MAX Efficiency® Stbl2™, Invitrogen™) were transformed with plasmid DNA by means of the heat shock procedure and selected by ampicillin. Pelleted bacteria were treated with the alkaline lysis procedure to purify the pK201/CAT plasmid DNA (pDNA) [30]. The quality of the preparation was checked by 1 % (w/v) agarose gel electrophoresis, and the concentration and purity assessed by determining the A260/280 and A260/320 ratios in UV-Vis spectrometry.

### Preparation of peptide/pDNA complexes

For the pDNA/peptide complexes, histone peptides were used at the concentration that allows the formation of electrically neutral complexes, according to both the negative to positive charges ratio (N/P) and the respective molar masses of the two species. Briefly, the volumes of the plasmid and the peptides calculated according to the desired concentrations were mixed in a sterile tube. After an incubation for 1 h at 37 ° C, the mixture were diluted to the final volume of coating in TBS, EDTA 10 mM (pH 7), and loaded on the plates for ELISA.

### Electromobility shift assay

For the electromobility shift assay (EMSA), pK201/CAT was linearized by EcoRI digestion. Linearized plasmid (5 μg) was incubated with histone peptides H2B(107-125), H4(56-71), H4(7-23), H4(31-50), H4(14-34) and its scramble form, H4(21-37), MAP-Cys-[H4(14-34)], and H4(14-22) at the previously established ratio. The amounts used were, respectively, 2.0, 2.38, 1.85, 1.1, 1.28, 1.60, 1.98, 1.68, 1.08, and 1.60 μg, with 1.65 μg of the control FcεRIα(46-65) peptide. After 1 h incubation at room temperature, the mixtures were loaded on 1.0 % agarose gel in TAE Tris-acetate-EDTA (TAE) buffer for 1.5 h along with an equal amount of non-restricted pDNA, restricted but non-complexed pDNA, and 2 μl 1 kb ladder (Bio Rad EZ load 1 kb). The gel was then stained with 5 μl ethidium bromide in 100 ml TAE buffer for 20 min. After a brief destaining step, the ethidium bromide fluorescence signal following UV irradiation was acquired by means of the VersaDoc Imaging System and QuantityOne analysis software (Bio-Rad, Hercules, CA, USA).

### Detection of anti-pDNA/peptide antibodies

Anti-pDNA/peptide antibodies were detected in the sera by ELISA. Briefly, the pPK201/CAT–H4(14-34) complex prepared as described above was diluted in TBS-EDTA 10 mM buffer (pH 7) at the final concentrations of 20 μg/ml for the pDNA and 6.37 μg/ml for the peptide, added to polystyrene plates (Nunc MaxiSorp F96; Nunc, Roskilde, Denmark), and incubated overnight at 4 °C. Saturation was carried out with PBS containing 3 % bovine serum albumin (BSA) for 45 min at room temperature. Sera diluted 1:250 (dilution offering the best discrimination between normal and pathological sera) in PBS containing 1 % BSA and 0.05 % Tween 20 were incubated in duplicate on the plates for 3 h at room temperature. After washing with PBS–0.1 % Tween, anti-human IgG alkaline phosphatase-conjugated antibody (Sigma-Aldrich, St Louis, MO, USA) was added to the wells, and the plates were incubated for 2 h at room temperature. Alkaline phosphatase activity was revealed with *p*-nitrophenyl phosphate in 50 mM Na_2_CO_3_/NaHCO_3_ (pH 9.6). Results were expressed as arbitrary units on the base of calibration curves set up by means of an internal standard.

### Detection of anti-pDNA antibodies

Anti-pDNA antibodies were detected by ELISA as previously described [31]. Briefly, polystyrene plates (Nunc MaxiSorp F96; Nunc, Roskilde, Denmark) were pre-coated for 1 h with polylysine 50 μg/ml, washed with TBS, and then coated with 20 μg/ml of the pDNA in TBS 10 mM EDTA. After overnight incubation at 4 °C, the residual positive charges of lysine were blocked by the addition of polyglutamate 50 μg/ml in TBS 10 mM EDTA. The plates were again washed with TBS, and saturation was carried out with PBS with 3 % BSA for 45 min at room temperature. Sera diluted in PBS with 1 % BSA 0.05 % Tween (diluting buffer) were incubated in duplicate on the plates for 3 h at room temperature. After washing with PBS–0.1 % Tween, anti-human IgG alkaline phosphatase-conjugated antibody (Sigma-Aldrich) was added to the wells, and the plates were incubated for 2 h at room temperature. Alkaline phosphatase activity was revealed with *p*-nitrophenyl phosphate in 50 mM Na_2_CO_3_/NaHCO_3_ (pH 9.6). Results were expressed as arbitrary units on the base of calibration curves set up by means of an internal standard.

### Detection of anti-dsDNA antibodies

Anti-dsDNA antibodies were detected according to manufacturer’s instructions by a commercially available ELISA (Aeskulisa dsDNA IgG, Aesku Diagnostic, Wendelsheim, Germany), based on a human recombinant dsDNA source as antigen bound to microwells. The values were expressed as IU/ml.

### Statistical analysis

Quantitative variables were compared between groups using Mann Whitney *U* test. The frequency of different clinical parameters was compared between anti-DNA-positive and anti-DNA-negative using the Fisher exact test. Sensitivity and specificity were evaluated by means of receiving operating characteristic (ROC) curves. Correlations were determined using Spearman Rank correlation coefficient. *P* < 0.05 was considered as significant; Prism 4 for Windows (GraphPad Software Inc.) was used for the analysis. Concordance was analyzed by means of the Cohen K coefficient.

## Results

### Histone peptides interact with plasmid DNA

Since histone H4 is known to interact with DNA, we explored the ability of H4 peptides to form complexes with DNA. With this aim, we synthesized linear peptides either containing or not containing the consensus sequence KRHRK for DNA binding (Table 1), and tested their ability to form complexes with plasmid DNA.

**Fig. 1:**
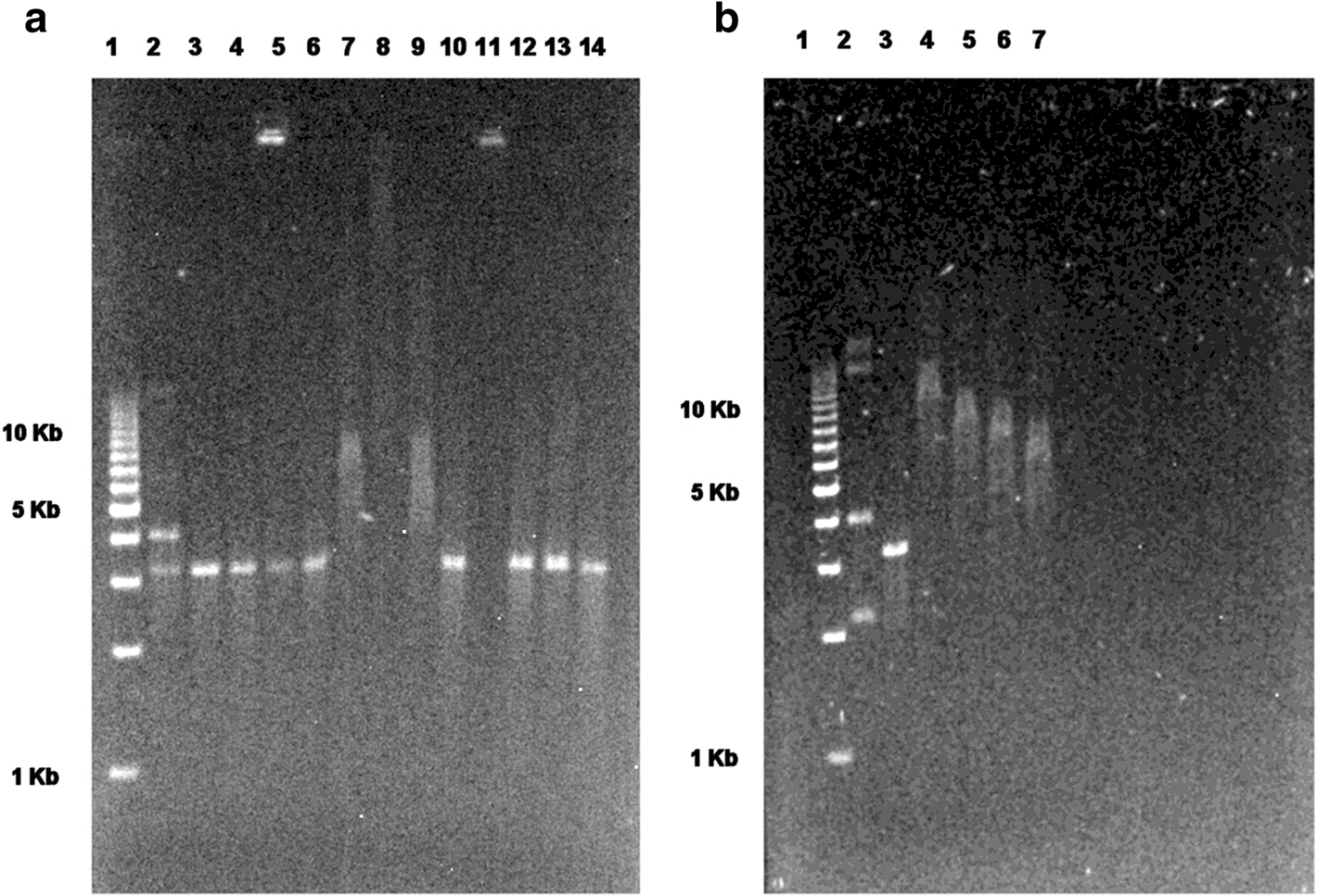
Histone 4 peptides interacting with plasmid DNA. Peptides were pre-incubated with plasmid DNA, linearized by means of EcoRI digestion, and the mix was loaded on agarose gel. The H4(14-34) and H4(14-22) peptides, but not H2b and control peptides, retard the migration of linearized plasmid DNA (**a**). The H4(14-34) peptide retards the migration of plasmid DNA in a concentration-dependent manner (**b**). **a**. Lane 1: MW marker 1 Kbp; Lane 2: pPK201/CAT, unrestricted; Lane 3: pPK201/CAT, restricted; Lane 4: restricted pPK201/CAT + H2B(107-125); Lane 5: restricted pPK201/CAT + H4(56-71); Lane 6: restricted pPK201/CAT + H4(76-91)-Gly; Lane 7: restricted pPK201/CAT + H4(7-22); Lane 8: restricted pPK201/CAT + H4(31-50); Lane 9: restricted pPK201/CAT + H4(14-34); Lane 10: restricted pPK201/CAT + H4(21-37); Lane 11: restricted pPK201/CAT + MAP-Cys-[H4(14-34)]4; Lane 12: restricted pPK201/CAT + H4(14-22); Lane 13: restricted pPK201/CAT + scramble H4(14-34); Lane 14: restricted pPK201/CAT + FcεRIα(46-65). **b**: Lane 1: MW markers 1 Kbp; Lane 2: PK plasmid, unrestricted; Lane 3: PK plasmid, restricted; Lane 4: PK plasmid, restricted + H4 peptide, 5 μg; Lane 5: PK plasmid, restricted + H4 peptide, 2.5 μg; Lane 6: PK plasmid, restricted + H4 peptide, 1.25 μg; Lane 7: PK plasmid, restricted + H4 peptide, 0.625 μg

The H4 peptides, as well as control peptides, were pre-incubated with pPK201/CAT DNA linearized by EcoRI digestion, and the mix was loaded on agarose gel. H4(14-34) and H4(7-22), but not other H4 sequences or control peptides including a peptide of similar charge, H2b(107-125), retarded the migration of linearized plasmid DNA in a concentration-dependent manner (Fig. 1a and b). H4(14-34) and H4(7-22) peptides were thus selected for further experiments.

### Peptide/DNA complexes as a probe to detect antibodies in sera

pPK201/CAT and H4(14-34) were preincubated at 37 °C for 1 h and then diluted in the final coating volume and added to polystyrene plates. Sera from NHS, disease controls (DC), and SLE patients were incubated on the plates coated with the peptide/plasmid complex, and the binding was detected by anti-IgG antiserum.

**Fig. 2:**
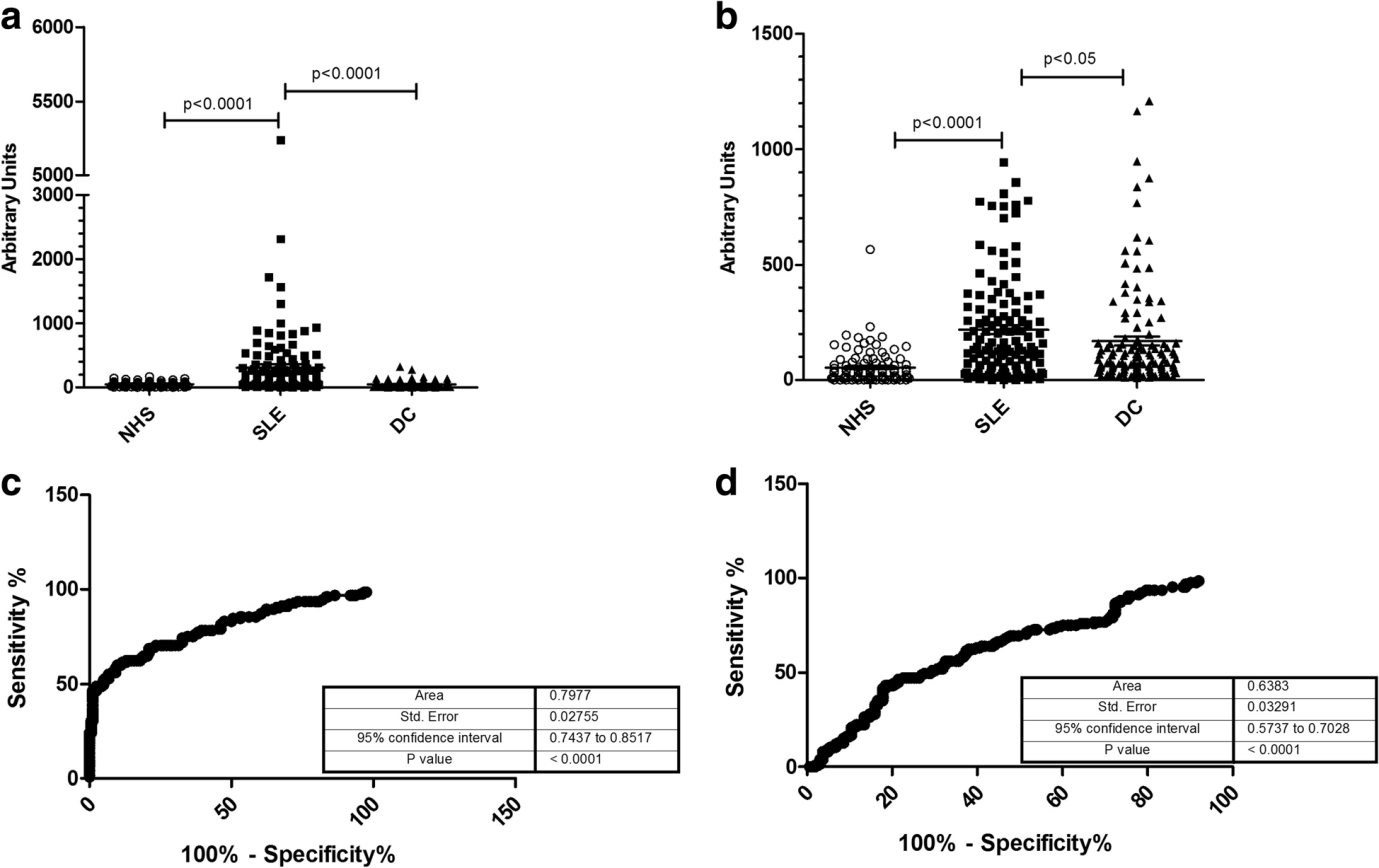
PK/H4 as a probe to detect antibodies in sera. PK/H4 complexes or pDNA (20 μg/ml) were used to coat polystyrene plates; diluted sera were incubated on plates and bound antibodies detected by anti-IgG antibodies. The PK/H4 complexes (**a**), but not the uncomplexed pDNA (**b**), are able to discriminate normal healthy subjects (*NHS*), systemic lupus erythematosus patients (*SLE*), and disease controls (*DC*). Sensitivity and specificity evaluated by the ROC curves are reported in **c** PK/H4 complex and **d** uncomplexed pDNA

The results indicate that PK201/H4(14-34) complexes discriminate between SLE patients and NHS or DC (Fig. 2a). In fact, 70 of 125 SLE sera contain anti-PK/H4 antibodies, as compared with 10 of 169 DC. No binding was detected to solid phase peptide (data not shown). On the contrary, SLE sera and also disease controls bind to solid phase PK201/CAT (Fig. 2b). The ROC curve confirms the better sensitivity and specificity of the assay employing the peptide DNA complex as compared with pDNA alone (Fig. 2c and d). Data on the binding of disease controls to pDNA and PK201/H4(14-34) are reported in Additional file 1 (Table S1).

In similar experiments performed with peptide H4(7-22), a lower number of SLE sera bound to the PK201/H4(7-22) complex compared to the PK201/H4(14-34) (35 % versus 55 %, data not shown); thus, further experiments were performed with H4(14-34) only.

**Fig. 3:**
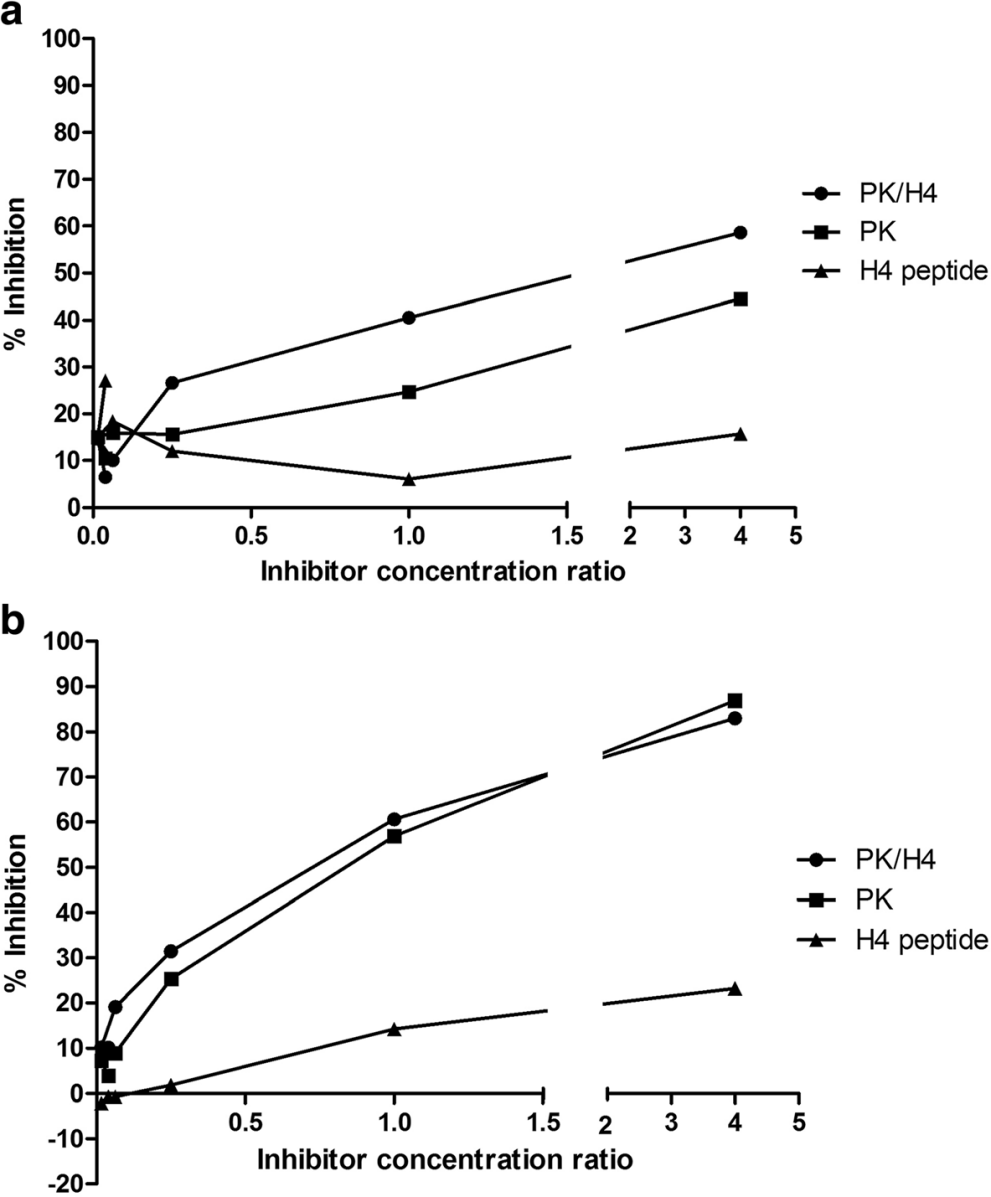
Specificity of anti-DNA antibodies by inhibition ELISA. SLE serum A (**a**) or serum B (**b**) were incubated with 1:4 dilutions of the PK/H4 complex, plasmid DNA (*PK*) or histone 5 (*H4*) peptide and then transferred to PK/H4 complex-coated plates. Results are expressed as percent inhibition of the binding observed in the absence of inhibitors [100 – (OD of inhibitor/OD of buffer) × 100]. A higher inhibition is obtained with PK/H4 complexes in the case of serum A, while in serum B complex or plasmid DNA pre-incubation results in identical inhibition. No inhibition is obtained with H4 peptide

To better define the specificity of serum antibodies, we performed inhibition assays by preincubating sera with PK/H4(14-34), plasmid DNA, or H4 peptide before transferring to PK/H4(14-34)-coated plates. In some sera, pre-incubation with PK/H4 or PK alone similarly inhibited the binding to solid phase PK/H4; in others, a higher inhibition is obtained with PK/H4, indicating the presence of antibodies preferentially reactive with the complex. H4 peptide does not exert any inhibition. Representative examples are given in Fig. 3. These results suggest that the epitopes recognized by serum antibodies reactive in the assay reside on DNA or on the complex, but not on the H4 peptide.

### Correlation with other assays for anti-dsDNA antibody detection

**Fig. 4:**
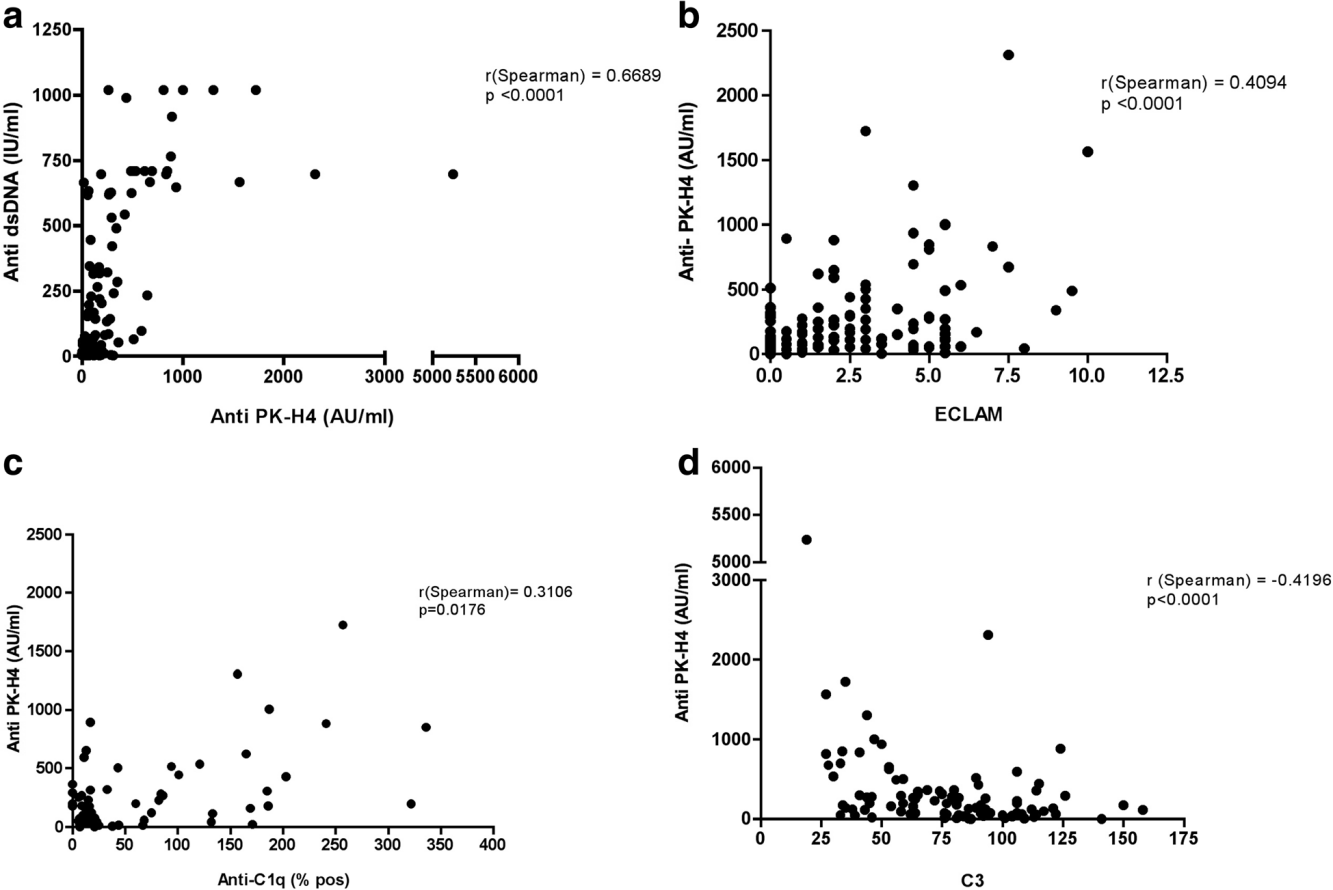
Diagnostic properties of PK/H4 complexes: correlations with Aeskulisa anti-dsDNA assay, ECLAM, anti-C1q, and C3. The amount of IgG specific for the PK/H4 complex are correlated with the level of anti-dsDNA antibodies detected by Aeskulisa (**a**), with the ECLAM score (**b**; r = 0.4094, *p* < 0.0001), and with anti-C1q autoantibodies titers (**c**; r = 0.3106; *p* = 0.0176), and inversely correlated with complement C3 levels (**d**; r = –0.4196; *p* < 0.0001). *C1q* complement component 1q, *ECLAM* European Consensus Lupus Activity Measurement, *H4* histone 4, *PK* plasmid DNA

A good correlation is observed with anti-dsDNA antibodies detected by Aeskulisa (Fig. 4a) but the results obtained with the two assays are not completely overlapping. If the vast majority of sera are either positive or negative on both tests, 26 sera are reactive only with dsDNA and 9 with PK/H4 only. Similar data are obtained comparing the PK/H4 assay with CLIF: 28 sera are reactive only with PK/H4 and 15 only by CLIF. The analysis of concordance is reported in Table 2.

**Table 2:** Analysis of concordance between the different assays for anti-dsDNA detection

	Anti-H4/PK vs Anti-dsDNA	Anti-H4/PK vs CLIF	Anti-dsDNA vs CLIF
Concordance	0.684	0.643	0.611
Cohen K	0.317	0.314	0.289

Cohen K values: 0.21–0.40 indicates modest concordance; 0.41–0.60 indicates moderate concordance; 0.61–0.80 indicates substantial concordance. *CLIF Crithidia Luciliae* immunofluorescence, *H4* histone 4, *PK* plasmid DNA

### Anti-PK/H4 antibodies, disease activity, and disease manifestations

The amount of IgG specific for the PK/H4 complex are correlated with the ECLAM score (r = 0.4094, *p* < 0.0001; Fig. 4b) and with anti-C1q autoantibody titers (r = 0.3106, *p* = 0.0176; Fig. 4c), and inversely correlated with complement C3 levels (r = –0.4196, *p* < 0.0001; Fig 4d). In this cohort of patients, the ECLAM score is also correlated with the amount of anti-pDNA antibodies (r = 0.51, *p* < 0.0001) or anti-dsDNA antibodies detected by Aeskulisa (r = 0.51, *p* < 0.0001).

The relationship between the presence and levels of anti-PK/H4 antibodies and the most frequent manifestations of the disease was then analyzed, in comparison with the assays for anti-dsDNA detection. The assays were able to detect antibodies in patients affected by arthritis or skin involvement or nephritis differently. In fact, the mean levels of antibodies were significantly higher in SLE patients with nephritis and arthritis when detected by anti-dsDNA Aeskulisa (*p* = 0.02 and *p* = 0.005, respectively), and in patients with arthritis and skin involvement when detected by PK/H4 (*p* = 0.007 and *p* = 0.02, respectively). Taking into account the frequency of positive results in the different groups, patients with skin involvement were more frequently positive with Aeskulisa (*p* = 0.048), patients with arthritis with PK/H4 (*p* = 0.048), and patients with nephritis with CLIFT (*p* = 0.039).

## Discussion

The detection of anti-dsDNA antibodies is a crucial issue in the diagnosis of SLE, especially in the early stages of the disease when the clinical manifestations and laboratory tests may not allow us to correctly classify the patient. At such a stage, overdiagnosis may lead to inappropriate treatment, while underdiagnosis may delay the use of drugs able to control the disease.

Despite the great relevance of anti-dsDNA antibodies and the technical progresses of the past years, the measurement of these antibodies in clinical practice still represents a challenge. The Farr assay is considered the gold standard in anti-dsDNA detection because of its high specificity and sensitivity and, mainly, for its high correlation with disease activity. However, the use of radioactive material and the short shelf life of radiolabeled DNA limits its use. CLIFT is a very specific assay with a high predictive value for the diagnosis of SLE, but is characterized by a low sensitivity and is not very suitable for the follow-up of patients. A wide number of solid phase assays have been developed, employing genomic or plasmid DNA in different assay formats, and endowed with different sensitivity and specificity; however, their diagnostic performances are not always optimal.

It has been suggested that the ability of CLIFT to detect SLE-specific anti-DNA antibodies may reside in the structural features of *Crythidia* DNA. Protozoan kinetoplast DNA contains minicircles characterized by 18 runs of 4–6 adenine in 200 bp; each run contributes to the global bending as the periodicity of the runs (1 every 10 bp) is in phase with the helix periodicity [18]. DNA bending is known to be related with nucleosome formation [32] and the PK201/CAT plasmid has the highest efficiency of interaction with core histones when compared with similar length plasmids not containing minicircles [33].

We thus selected the PK201/CAT plasmid and used it as a probe to measure antibodies in sera. The plasmid DNA on the solid phase allows us to detect anti-dsDNA antibodies, but the assay specificity is unsatisfactory when disease controls are taken into account. To better mimic nucleosomal DNA, we analyzed the interaction of plasmid DNA with basic peptides derived from histones.

Core histones possess very basic amino terminal regions that interact with nucleosomal DNA. In the case of H4, the amino acid stretch involved in the interaction with the double helix and responsible for DNA kinking is represented by the highly charged sequence 16-23 [19]. X-ray crystallography of DNA complexes with core histones at a resolution of 1.9 Angstrom depicts with higher detail the interaction of H4 with DNA: hydrogen bonds are formed between DNA and Q27, T30, K31, P32, and R36 residues, while R17 and R19 associate with phosphate groups [20].

Among the H4 peptides we studied, only those containing the consensus sequence form a stable complex with plasmid DNA. A sequence of similar charge from H2b does not interact directly with DNA, according to crystallographic studies [34], and was not able to retard the migration of plasmid DNA.

The stable interaction of H4 peptide with plasmid DNA has allowed us to build a simple and reliable assay that does not require multistep procedures such as pre-coating or DNA labeling, all potential sources of interferences.

By analyzing the epitopes expressed on the complex by means of inhibition assays, we obtained direct evidence for the existence of multiple epitopes, some residing on the DNA moiety and some formed by the interaction of DNA with the peptide. The assay can thus detect autoantibodies binding either DNA or the DNA/peptide complex. This ability to measure antibodies of different specificity, including anti-dsDNA, is depicted by the good correlation, but low concordance, with the “classical” assays for anti-dsDNA antibodies.

Combined with clinical and serological data, this assay can contribute to a correct diagnosis and staging of SLE patients. The high specificity of this test allows the differential diagnosis of SLE from other connective tissue disorders, as only 5.9 % of disease control sera contain very low levels of anti-PK/H4 antibodies. In the disease control group, both rheumatoid factor-positive and -negative RA sera were tested, thereby excluding any interference of rheumatoid factors in the assay. It is also relevant that the group of disease controls includes a high number of UCTD patients that share clinical and serological manifestations with definite connective tissue diseases but who do not fulfill any of the existing classification criteria [26]. Thus, the major novelty of the assay is its ability to discriminate SLE from disorders that may clinically or serologically resemble SLE.

The detection of anti-PK/H4 antibodies also gives reliable information on disease activity, as indicated by the positive correlation with the ECLAM score and the inverse correlation with complement levels. Moreover, the level of anti-PK/H4 antibodies is highly correlated with the level of anti-C1q antibodies that are considered a reliable biomarker of active disease, especially at the renal level [35–37].

As mentioned above, comparing the results obtained with the CLIF test, anti-dsDNA Aeskulisa, and anti-PK/H4, we observed a limited concordance, suggestive of the presence in SLE sera of different populations of anti-DNA antibodies which are differentially reactive in the different assays. Similar observations have been previously reported and led to the recommendation that more than one assay should be used to better depict the repertoire of anti-DNA present in a serum since no assay is able to detect pathogenic antibodies only [9, 38]. When the presence and amounts of anti-PK/H4 antibodies were related with disease manifestations, we found that patients with arthritis and active skin disease had higher levels of antibodies. CLIFT is more often positive in patients with active renal disease, as previously found [12–14], and dsDNA detects antibodies in patients with skin or renal involvement.

## Conclusions

In conclusion, these clinico-serological correlations suggest that different assays can detect anti-dsDNA antibodies of different pathogenic potential. The PK/H4 complex may represent a novel tool to increase the variety of autoantibodies that can be measured, complementing the assays currently used for the detection of anti-DNA antibodies.

## Additional file

Additional file 1: Table S1. Number of positive sera in the different control diseases. (DOC 31 kb)

## Abbreviations

APS: Anti-phospholipid syndrome
BSA: Bovine serum albumin
C1q: Complement component 1q
CLIFT: *Crithidia Luciliae* immunofluorescence test
DC: Disease controls
DM: Dermatomyositis
ECLAM: European Consensus Lupus Activity Measurement
EMSA: Electromobility shift assay
IgG: Immunoglobulin G
IIM: Idiopathic inflammatory myopathies
H4: Histone 4
NHS: Normal healthy subjects
PK: PK201/CAT plasmid
PM: Polymyositis
PMR: Polymyalgia rheumatica
PsA: Psoriasic arthritis
RA: Rheumatoid arthritis
ROC: Receiving operating characteristic
SjS: Sjogren syndrome
SLE: Systemic lupus erythematosus
SSc: Systemic sclerosis
TAE: Tris-acetate-EDTA buffer
UCTD: Undifferentiated connective tissue disease
