# Supplementary material for: A novel DNA/histone H4 peptide complex detects autoantibodies in systemic lupus erythematosus sera
Source: Arthritis Res Ther. 2016 Oct 4;18:220. doi: 10.1186/s13075-016-1117-8 (PMC5050916; doi:10.1186/s13075-016-1117-8)
Supplement: Additional file 1: Table S1. — Number of positive sera in the different control diseases. (DOC 31 kb) [file 13075_2016_1117_MOESM1_ESM.doc]

| **Control disease** | **PK-H4** | **pDNA** | **Aeskulisa** |
| --- | --- | --- | --- |
| RA | 0/40 | 0/40 | 0/40 |
| IIM | 2/30 | 0/30 | NT |
| UCTD | 6/29 | 9/29 | 19/29 |
| SSc | 0/29 | 9/29 | 2/14 |
| SjS | 1/27 | 6/27 | 7/12 |
| APS | 0/5 | 0/5 | 3/5 |
| PsA | 1/4 | 1/4 | 0/4 |
| PMR | 0/3 | 0/3 | 0/3 |
| Systemic vasculitis | 0/2 | 0/2 | 0/2 |

Table S1. Number of positive sera in the different control diseases
